# Investigating the links between diagnostic uncertainty, emotional exhaustion, and turnover intention in General Practitioners working in the United Kingdom

**DOI:** 10.3389/fpsyt.2022.936067

**Published:** 2022-07-26

**Authors:** Anli Yue Zhou, Salwa S. Zghebi, Alexander Hodkinson, Mark Hann, Christos Grigoroglou, Darren M. Ashcroft, Aneez Esmail, Carolyn A. Chew-Graham, Rupert Payne, Paul Little, Simon de Lusignan, Sudeh Cherachi-Sohi, Sharon Spooner, Andrew K. Zhou, Evangelos Kontopantelis, Maria Panagioti

**Affiliations:** ^1^National Institute for Health and Care Research (NIHR) School for Primary Care Research, Division of Population Health, Health Services Research and Primary Care, Faculty of Biology, Medicine and Health, University of Manchester, Manchester, United Kingdom; ^2^NIHR Greater Manchester Patient Safety Translational Research Centre, Faculty of Biology, Medicine and Health, University of Manchester, Manchester, United Kingdom; ^3^Institute for Health Policy and Organisation (IHPO), Faculty of Biology, Medicine and Health, Manchester Academic Health Science Centre, University of Manchester, Manchester, United Kingdom; ^4^Centre for Pharmacoepidemiology and Drug Safety, Division of Pharmacy and Optometry, University of Manchester, Manchester, United Kingdom; ^5^School of Medicine, Keele University, Newcastle, United Kingdom; ^6^Centre for Academic Primary Care Population Health Sciences, Bristol Medical School, University of Bristol, Bristol, United Kingdom; ^7^Primary Care, Population Sciences and Medical Education, University of Southampton, Southampton, United Kingdom; ^8^Medical Sciences Division, Nuffield Department of Primary Care Health Sciences, University of Oxford, Oxford, United Kingdom; ^9^Royal College of General Practitioners Research and Surveillance Centre, London, United Kingdom; ^10^Division of Informatics, Imaging and Data Sciences, Faculty of Biology, Medicine and Health, Manchester Academic Health Science Centre, University of Manchester, Manchester, United Kingdom

**Keywords:** burnout, diagnostic uncertainty, presenteeism, work-life balance, general practice, General Practitioners

## Abstract

**Background:**

General Practitioners (GPs) report high levels of burnout, job dissatisfaction, and turnover intention. The complexity of presenting problems to general practice makes diagnostic uncertainty a common occurrence that has been linked to burnout. The interrelationship between diagnostic uncertainty with other factors such as burnout, job satisfaction and turnover intention have not been previously examined.

**Objectives:**

To examine associations between diagnostic uncertainty, emotional exhaustion (EE), depersonalization (DP), job satisfaction, and turnover intention in GPs.

**Methods:**

Seventy general practices in England were randomly selected through the Oxford-Royal College of General Practitioners Research and Surveillance Centre (RCGP-RSC). A total of 348 GPs within 67 these practices completed a 10-item online questionnaire which included questions on GP characteristics, work-life balance, job satisfaction, sickness presenteeism, diagnostic uncertainty, turnover intention as well as EE and DP. Associations between diagnostic uncertainty and each of EE, DP, job satisfaction, and turnover intention were evaluated in multivariate mixed-effect ordinal logistic regressions whilst adjusting for covariates, to account for the correlation in the three outcomes of interest.

**Results:**

Almost one-third of GPs (*n* = 101; 29%) reported experiencing >10% of diagnostic uncertainty in their day-to-day practice over the past year. GPs reporting greater diagnostic uncertainty had higher levels of EE [OR = 3.90; 95% CI = (2.54, 5.99)], job dissatisfaction [OR = 2.01; 95% CI = (1.30, 3.13)] and turnover intention [OR = 4.51; 95% CI = (2.86, 7.11)]. GPs with no sickness presenteeism had lower levels of EE [OR = 0.53; 95% CI = (0.35, 0.82)], job dissatisfaction [OR = 0.56; 95% CI = (0.35, 0.88)], and turnover intention [OR = 0.61; 95% CI = (0.41, 0.91)].

**Conclusion:**

Diagnostic uncertainty may not only negatively impact on the wellbeing of GPs, but could also have adverse implications on workforce retention in primary care.

## Introduction

Burnout in General Practitioners (GPs) is a widely recognized problem in primary care (1) and it is estimated that more than 50% of GPs working in the UK have reported moderate or high levels of burnout (2). GPs have been reporting high workload pressures and demands in their day to day practice and have been found to be at high risk of burnout compared to other specialties (2). Burnout in GPs has been linked with low job dissatisfaction, sickness presenteeism and diagnostic uncertainty in primary care and therefore can have major adverse implications on the primary care delivery (3–7). Furthermore, GPs who have been found to be at high risk of burnout are more likely to report intention to quit (1, 2, 8), which in turn may have wider implications on workforce retention in an already stretched primary care workforce.

Diagnostic uncertainty has been defined as a “*subjective perception of an inability to provide an accurate explanation of the patient's health problem*.” (9) and is a major challenge in primary care where GPs are often faced with making a diagnosis based on early symptoms, of a wide range of diagnostic possibilities at the point of patient presentation and with reduced and/or delayed access to diagnostic tests (10) and GPs have been found to experience one of the highest levels of uncertainty compared to other medical specialties (10, 11).

Diagnostic uncertainty can contribute to medical errors and are more likely to occur when doctors are unfamiliar with the patient and when presentations are non-specific (12). Diagnostic errors have been found to be the most common cause of avoidable significant harm (13) and medical errors due to diagnostic uncertainty can have serious implications for patients, medical practitioners and healthcare systems (12), leading to poorer patient outcomes, medical litigation, defensive medicine and delivery of care, as well as increases in healthcare costs (14). Although GPs encounter uncertainty in their day-to-day practice, very few studies have explored the association between diagnostic uncertainty, burnout, job satisfaction, sickness presenteeism and turnover intention (3, 4).

The overall sustainability of the primary care system does depend on the function and wellbeing of its workforce and therefore investigating the associations between diagnostic uncertainty, job satisfaction, turnover intentions, burnout subcomponents such as Emotional Exhaustion (EE) and Depersonalization (DP), and may provide insight into ways to mitigate burnout in a workforce that is already under immense pressure. This in turn could lead to a greater understanding of factors that have not been previously explored in conjunction with each other, with the aim that this could improve the quality of both physician wellbeing and patient care.

## Methods

### Study design

This was a cross-sectional study using a self-reported online questionnaire involving 70 randomly selected general practices in England. Participants were recruited through the Oxford-Royal College of General Practitioners (RCGP) Research Survey Centre (RSC) between December 2019 to March 2020, in a period prior to the COVID-19 pandemic (15).

All GPs who were working in the 70 selected practices were considered eligible to participate in the study.

### Study measures

An online 10-item questionnaire link was sent to participating GP practices through the RCGP RSC using the survey monkey platform (16). This included three items on key GP and practice characteristics (full-time equivalent [FTE] status of the responder, sum of FTE of all GPs in the practice, and age). There were five validated items on % of diagnostic uncertainty, overall job satisfaction, sickness presenteeism over the past 12 months, turnover intention within the next 5 years and work-life balance (see Supplementary Material) (17–20). A validated abbreviated scale of the Maslach Burnout inventory consisting of 2 items (EE and DP) was used (21).

The RSC provided anonymized data on GP gender, practice list size, practice-level National Health Service (NHS) region, practice location, as well as the 2019 Index of Multiple Deprivation (IMD) quintiles which is a composite area deprivation score which includes seven domains [income, employment, education, health, living environment, barriers to housing, and services and crime (22)].

### Sample selection

The GP survey was intended to reach 350–400 GPs across 70 different practices from the total nationally representative pool of general practices in RSC consistent with our funding availability. Each participating GP received a £20 payment to their GP practice. The practices were selected randomly and the distribution of the survey was done using random sampling by the RSC and avoided selection bias by practice. The random sample was representative of the distribution of GP practices in England.

### Data analysis

Over 99% of data were provided for all wellness factors and variables and the remaining missing values were imputed using the R package ‘MICE: Multivariate Imputation by Chained Equations’ (23).

Descriptive statistics were then used to assess the baseline characteristics of the GPs involved. Differences between wellbeing factors were assessed with the Kruskal-Wallis H test (24) and polychoric correlations was estimated between wellbeing factors (25).

We used a multivariate mixed-effect ordinal logistic model, fitted using *mvord* package in R software (26), to account for the correlation in the four outcomes (EE, DP, job dissatisfaction, and turnover intention). The following covariates were included in the model: GP demographics (age, FTE, and gender), GP practice characteristics (NHS region, IMD quintile, and list size) and wellbeing factors (work-life balance, diagnostic uncertainty, and sickness presenteeism). A mixed-effect model was used to assess for GP clusters within practices.

Clinical expertise of the co-authors (MP and MH) and data availability drove the decision as to which variables were to be included in the models. Variance inflation factors were used to examine for multicollinearity and scores below 4 were consider as uncorrelated (27). The statistical analyzes was produced through R software to undertake the multivariable mixed-effect ordinal logistic regression (28).

## Results

### Demographics and descriptive results

The survey reached 67 practices and a total of 348 completed surveys were received. Baseline characteristics are presented in Table 1. Twenty-two percent of GPs reported high levels of EE and 16% reported high levels of DP. Over 40% of GPs reported poor work-life balance and nearly 75% of GPs reported sickness presenteeism at work. Only 60% of GPs reported job satisfaction in their current job roles and about one third of GPs reported no intention to quit over the next 5 years. All wellbeing factors when statistically compared were significantly different, with the exception being diagnostic uncertainty and DP [χ^2^(4) = 7.39, *p* = 0.117] and diagnostic uncertainty and turnover intention [χ^2^(4) = 7.85, *p* = 0.097].

**Table 1:** Baseline characteristics of the recruited 348 General Practitioners.

**Variable**	***N*** (%)^*^
Number of general practices	67
Practice-level index of multiple deprivation (IMD)	
• Quintile 1 (least deprived)	46 (13.2)
• Q2	45 (12.9)
• Q3	56 (16.1)
• Q4	68 (19.5)
• Q5 (most deprived)	62 (17.8)
Not available	71 (20.4)
NHS region	5 (1.4)
• London	31 (8.9)
• Midlands And East	31 (8.9)
• North	110 (31.6)
• South	131 (37.6)
Not available	71 (20.4)
Age, mean (±SD)	45 (±8.5)
Age	
• <40 years	100 (28.7)
• 40–49 years	139 (39.9)
•≥50 years	109 (31.3)
Gender	
• Male	152 (43.7)
• Female	196 (56.3)
Years worked in practice	
• <1 year	26 (7.5)
• 1–5 year	101 (29.0)
• 6–10 year	63 (18.0)
• 11–20 year	91 (26.2)
•>20 years	67 (19.3)
FTE category	
• 0–0.500	81 (23.3)
• 0.501–0.750	120 (34.5)
• 0.751–1.000	146 (42.0)
• Not available	1 (0.3)
FTE, mean (±SD)	0.737 (±0.22)
**Wellbeing factors**	
Diagnostic uncertainty	
• 0–5%	124 (35.6)
• 6–10%	123 (35.3)
• ≥11%	101 (29.0)
Frequency of Emotional exhaustion (EE)	
• Never	34 (9.8)
• A few times a year or less	108 (31.0)
• Once a month or less	58 (16.7)
• A few times a month	70 (20.1)
• Once a week	31 (8.9)
• A few times a week	38 (10.9)
• Daily	9 (2.6)
Frequency of Depersonalization (DP)	
• Never	68 (19.5)
• A few times a year or less	111 (31.9)
• Once a month or less	60 (17.2)
• A few times a month	54 (15.4)
• Once a week	13 (3.7)
• A few times a week	34 (9.8)
• Daily	8 (2.3)
Turnover intention within the next 5 years	
• None	115 (33.1)
• Slight	119 (34.0)
• Moderate	48 (13.8)
• Considerable	34 (9.8)
• High	32 (9.2)
Work-life balance	
• Strongly agree/ Agree	133 (38.2)
• Neutral	73 (22.0)
• Disagree/Strongly Disagree	142 (40.8)
Sickness presenteeism over the past 12 months	
• Never	94 (27.0)
• Once	111 (31.9)
•≥2 times	143 (41.1)
Job Satisfaction	
• Strongly agree	48 (13.8)
• Agree	162 (46.6)
• Neutral	73 (21.0)
• Disagree	51 (14.7)
• Strongly Disagree	14 (4.0)

* *Unless otherwise stated*.

The estimated correlations for the wellbeing factors ranged from small to moderate (0.109–0.591). The strongest correlation was shown between EE and turnover intention (ρ = 0.591) and the weakest correlation was between work-life balance and job satisfaction (ρ = 0.109; Table 2).

**Table 2:** Polychoric correlations of all GP wellness factors.

**EE**						
0.456	DP					
0.347	0.150	Sickness presenteeism				
0.238	0.144	0.096	Work-life balance			
0.497	0.164	0.283	0.110	Diagnostic uncertainty		
0.467	0.246	0.274	0.109	0.405	Job satisfaction	
0.591	0.295	0.346	0.210	0.503	0.429	Turnover intention

### Multivariate mixed-effect ordinal regression results

Table 3 summarizes the multivariate mixed-effect ordinal logistic regression results. High levels of diagnostic uncertainty (>10%) was found to be associated with increased odds for EE [OR 3.9 (95% CI, 2.54, 5.99)], increased odds for job dissatisfaction [OR 2.01 (95% CI 1.3, 3.13)] and increased odds for turnover intention [OR 4.51 (95% CI 4.51 (2.86, 7.11))]. In contrast, GPs who were ≥50 years were much less likely to report turnover intention [OR 0.54 (95% CI 0.33, 0.90)] compared to GPs <40 years of age.

**Table 3:** Multivariate mixed-effect ordinal regression model of the relationship between GP demographic, practice and wellbeing factors with burnout (emotional exhaustion, depersonalization), job dissatisfaction, and turnover intention.

**Variable**	**Emotional exhaustion** ^*^		**Depersonalization** ^*^		**Job dissatisfaction** ^*^		**Turnover intention** ^*^	
	**OR**	**95% CI**	**OR**	**95% CI**	**OR**	**95% CI**	**OR**	**95% CI**
Age^¥^	Reference = Up to 39 yrs.							
• 40–49 yrs.	1.22	0.81, 1.85	1.08	0.71, 1.62	1.39	0.96, 2.04	0.98	0.65, 1.49
• 50 yrs. and over	0.81	0.52, 1.26	0.83	0.52, 1.32	1.25	0.78, 2.00	**0.54**	**0.33, 0.90**
Gender:	Reference = Male							
- Female	0.81	0.58, 1.13	0.99	0.70, 1.39	**0.70**	**0.50, 0.99**	0.90	0.59, 1.36
Work-life balance:	Reference = Disagree/Strongly disagree work schedule leaves me with good work-life balance							
- Neutral	0.81	0.54, 1.20	0.89	0.60, 1.33	0.87	0.58, 1.32	0.96	0.61, 1.52
- Strongly agree/Agree	**0.67**	**0.45, 0.99**	0.81	0.53, 1.24	0.78	0.52, 1.15	0.82	0.54, 1.25
Diagnostic uncertainty:	Reference = 0 to 5% patients were difficult to diagnose							
- 6–10%	**2.04**	**1.24, 3.36**	1.32	0.80, 2.16	1.47	0.87, 2.47	**3.44**	**1.99, 5.92**
- Over 10%	**3.90**	**2.54, 5.99**	1.53	0.97, 2.40	**2.01**	**1.30, 3.13**	**4.51**	**2.86, 7.11**
Presenteeism:	Reference = Presented at work more than once with a sickness							
- Never	**0.53**	**0.35, 0.82**	0.78	0.49, 1.22	**0.56**	**0.35, 0.88**	0.66	0.42, 1.04
- Once	0.73	0.49, 1.10	0.88	0.58, 1.33	0.81	0.56, 1.18	**0.61**	**0.41, 0.91**
IMD Quintile:	Reference = IMD code 1 (least deprived)							
- 2	1.07	0.58, 1.97	1.11	0.60, 2.07	1.29	0.75, 2.23	1.55	0.86, 2.79
- 3	1.31	0.72, 2.39	0.91	0.52, 1.58	1.17	0.67, 2.05	1.59	0.87, 2.89
- 4	1.00	0.55, 1.84	0.71	0.42, 1.22	1.04	0.59, 1.82	1.20	0.68, 2.11
- 5 (most deprived)	0.79	0.45, 1.38	0.83	0.49, 1.41	0.77	0.45, 1.33	1.04	0.59, 1.81
FTE^¥^	1.58	0.82, 3.05	**2.36**	**1.30, 4.28**	**2.43**	**1.37, 4.33**	1.18	0.52, 2.71
List size^¥^	1.00	0.99, 1.00	1.00	0.99, 1.00	1.00	0.99, 1.00	0.99	0.99, 1.00
NHS Region:	Reference = North							
- London	0.65	0.13, 3.18	1.24	0.37, 4.11	0.44	0.09, 2.05	1.86	0.37, 9.39
- Midland and East	1.01	0.58, 1.78	1.08	0.53, 2.18	0.91	0.50, 1.67	0.86	0.47, 1.59
- South	0.80	0.55, 1.17	0.89	0.61, 1.29	0.98	0.66, 1.45	1.00	0.68, 1.47

* *Treated as ordinal responses (higher scores were unfavorable) and fitted using mvord package in R*.

¥ *Continuous variables*.

*FTE, full-time equivalent. The bold values indicate the statistically significant values*.

Over and above diagnostic uncertainty, GPs who did not report any episodes of sickness presenteeism (or reported only one episode) over the past 12 month had decreased odds for EE [OR 0.53 (95% CI 0.35, 0.82)], decreased odds for job dissatisfaction [OR 0.56 (95% CI 0.35, 0.88)] and increased odds of turnover intention [OR 0.61 (95% CI 0.41, 0.91)] compared to GPs who reported >1 episode. GPs reporting a good work-life balance had decreased odds for experiencing EE [OR 0.67 (95% CI 0.45, 0.99)]. GPs working longer hours (higher FTE) had over increased odds for higher DP [OR 2.36 (95% CI 1.30, 4.28)] and increased odds of being dissatisfied with their job [OR 2.43 (95% CI 1.37, 4.33)]. Gender, age, list size, NHS region and practice-level IMD were not found to be associated with EE, DP, or turnover intention. However, female GPs were marginally less likely to report job dissatisfaction. There was no evidence of multicollinearity in the set of covariates that was included in the model.

## Discussion

### Statement of principal findings

This study, using a national cohort involving 67 practices and 348 GPs, found that GPs reporting higher levels of diagnostic uncertainty had two to five times higher risk of EE, job dissatisfaction and turnover intention. Beyond diagnostic uncertainty, GPs reporting multiple episodes of sickness presenteeism were also found to have two times higher risk of EE, job dissatisfaction and turnover intention. GPs reporting poor work-life balance had 1.5 times higher risk of EE, whereas those working longer hours had 2 times higher risk of DP and job dissatisfaction. Characteristics such as age, gender, IMD quintile, NHS region, list size were not found to be associated with EE or DP.

### Meaning of the study: Possible mechanisms and clinical implications

Our study found a clear relationship between GPs experiencing high levels of diagnostic uncertainty (>10%) and EE, job dissatisfaction and turnover intention compared to GPs who experienced low levels of diagnostic uncertainty. Previous studies in primary care have focused on GP trainees which identified that an intolerance of uncertainty was associated with burnout (4) and this association was also found in emergency medicine trainees too (29). In our study, participants were fully qualified GPs who have a level of expertise in this specialty (64% with >6 years of working experience). The level of work experience has been suggested to modify perceptions of diagnostic uncertainty (10, 30), however, our study has demonstrated that diagnostic uncertainty continues to occur even in experienced GPs and can put GPs at higher risk of EE which could subsequently lead to burnout. One component of diagnostic uncertainty that was previously found to be predict burnout in emergency medicine physicians was related to concerns about bad outcomes (31) and diagnostic uncertainty has been previously linked to diagnostic errors in primary care (32).

Our study only assessed for the level of diagnostic uncertainty the GP had experienced, while future research could consider the reasons behind diagnostic uncertainty to help develop interventions to help GPs to embrace uncertainty in their day-to-day practice. Although studies have explored factors driving turnover intention in GPs such as workload, low job satisfaction and burnout (1, 33), diagnostic uncertainty and turnover intention in primary care have not been previously explored. This further highlights the need to understand the wider implications of diagnostic uncertainty on service delivery and workforce retention.

Our study identified that GPs reporting multiple episodes of sickness presenteeism was associated with higher levels of EE, job dissatisfaction, and turnover intentions and similar results have also been reflected previously in the literature (7, 34). Sickness presenteeism is common amongst senior doctors which could be related to having a higher threshold of recognizing illness in themselves, showing commitment to their workplace and reserving their sick leave for when their dependents are unwell (35). Furthermore, lower sickness presenteeism levels have also been found when perceived social support is available (36). This suggests there is a need to modify perceptions and attitudes around doctors' sickness behaviors in order to mitigate EE and potentially burnout and improve workforce retention in primary care as well the need to invest in organizational changes that will enable adequate resources in primary care and to make workloads to be more manageable (37).

GPs within our study who reported poor work-life balance were found to be at a higher risk of EE which is consistent with previous research on GPs (38). GPs have been found to have one of the higher rates of burnout and poor work-life balance compared to other medical specialties (39) and high work demands have led to GPs choosing to reduce working hours or retire early due to work pressures (40, 41). These findings suggest perceived poor work-life balance can not only lead to EE but also put additional pressures on a healthcare system that is already over-stretched. Although multiple factors that can negatively impact on primary care are known such as increasing workloads, service delivery demands, complaints and litigation (1, 41), our study shows that work-life balance remains a concern in GPs and effective interventions are required to improve the working lives of GPs.

Previous literature has suggested that older GPs aged >50 years reported high levels of intention to leave (42) which is in contrast to our results. Factors that have been linked to turnover intention in older GPs include perverse tax situation, early retirement being a viable option, alternative employment options, work-related factors such as workload and burnout (43, 44). It may have been possible that our GP cohort did not have early retirement or alternative employment as viable options or alternatively, may have been satisfied with their current role. Another possibility could be that GPs who did not report turnover intention may already be close to retirement age and did not wish to leave their current role. However, due to the nature of our study, we were not able to explore why turnover intention was lower in older GP participants, however, this could be an interesting area to understand in order to help explore strategies to support workforce retention, especially in light of the COVID-19 pandemic.

Our study did not find an association between practice level IMD and EE or DP which seems to contrast with previous research that has highlighted the complexity and demands of caring for patients in the context of high socioeconomic deprivation (45). Factors that could be driving GPs' workload and burnout include increased multimorbidity, increased emotional demands in managing perceived overwhelming patient demand, high prevalence of psychosocial problems and complex social challenges (45). Most studies on GPs working in socially deprived locations have been qualitative in nature (45, 46), and our study only had a small number of GPs (*n* = 62) working in the most deprived quintile, suggesting there is a need to quantify the relationship between social deprivation and GP burnout.

Within our study, DP was not found to be associated with diagnostic uncertainty or any demographic or practice factors except for FTE, with GPs working full-time were at higher risk of DP compared to those working fewer hours. Previous research has found that male doctors and career length has been associated with DP in primary care (47), however the study did not explore whether FTE or part-time showed any differences in DP. Increasing workloads and patient demands have been identified as one of the main job stressors in GPs (48) and therefore GPs working full-time may experience higher workloads and demands, and full-time GPs have reported to work an average of around 55 h per week (48). A previous study has found that for every additional 5 h of work worked beyond the 40 h week, the risk of burnout increases (49), however, this finding was only found in female doctors. Further research may be required to assess the relationship between FTE and burnout and to explore the relationships between FTE, working hours, burnout, and gender.

### Strengths and limitations of the study

Diagnostic uncertainty is not uncommon in primary care however, the strength of this study is that it has not only explored diagnostic uncertainty but specifically explored the link between diagnostic uncertainty, turnover intention, job dissatisfaction, and burnout in GPs across practices in England. Furthermore, our findings also highlight potential areas for future research that could not only have implications to GP wellbeing but also wider implications to GP workforce retention and primary care service delivery. However, this study has several limitations. First, this was a cross-sectional study and therefore causation cannot be determined. Secondly, although the survey was intended to reach over 700 GPs, however, we only received 348 complete responses which gives an overall response rate of <50%. The response rate in this study is similar to previous studies involving GPs (50–52), however, our results may only reflect the perspectives of participating GPs rather than all GPs working in practices across the UK. Furthermore, although we used validated items within our survey, however, our results are based on self-reported data on abbreviated measures and there could be a risk of recall bias especially if GPs are asked to recall information over the preceding 12 months. In our study, we assessed EE and DP as separate dependent variables, however, another potential consideration for future research could be to consider EE and DP together as a dependent variable Within our study, diagnostic uncertainty was found to be an important factor associated with emotional exhaustion, turnover intention and job satisfaction. The measure included in our study was developed using a comprehensive literature review, analyzes of medical legal claims and pilot tested through cognitive interviews (19) and therefore this was considered a suitable measure for diagnostic uncertainty in our survey which was designed to minimize survey fatigue. However, it is acknowledged that this measure may have limitations in its validity and more research is needed in this area to create a method to comprehensively assess diagnostic uncertainty as there is currently no gold standard to assess diagnostic uncertainty (9). Furthermore, the study was also conducted pre-COVID so the results may be less relevant given considerable changes in primary care following COVID-19 restrictions.

## Conclusion

Diagnostic uncertainty was found to be associated with EE, turnover intention and job dissatisfaction suggesting the need to understand not just how diagnostic uncertainty impacts individual GPs but also the wider negative implications of diagnostic uncertainty in the workforce planning and service delivery of primary care. Alongside diagnostic uncertainty, sickness presenteeism also needs to be considered and addressed while designing GP wellness and workforce retention remedies in primary care such as addressing workload, adequate resources and a supportive environment.

## Data availability statement

The datasets presented in this article are not readily available because the data is stored in a repository which is managed by the Royal College of General Practitioners Research and Surveillance Centre (RSC). Any requests to access the datasets should be directed to maria.panagioti@manchester.ac.uk.

## Ethics statement

The project was reviewed by the University of Manchester's research ethics committee IRAS ID: 330 268533. Written informed consent for participation was not required for this study in accordance with the national legislation and the institutional requirements.

## Author contributions

MP, PL, RP, CC-G, AE, and DA contributed to the conception of the study. SZ, AH, CC-G, MP, SL, PL, RP, CC-G, AE, and DA contributed to funding acquisition, design study, and data collection. SZ, AH, MH, EK, and MP contributed to the data analysis. AYZ, SZ, AH, MH, and MP contributed to data interpretation and visualization. AYZ drafted the original manuscript draft. SZ, AH, MH, CC-G, SC-S, SS, and AKZ contributed to specific sections in the manuscript. All authors contributed to the manuscript revision, read, and have approved the submitted version.

## Funding

This study was funded by the NIHR School for Primary Care Research (project 408) and the National Institute for Health and Care Research (NIHR) through the Greater Manchester Patient Safety Translational Research Centre (award number: PSTRC-2016-003). AH was funded by his NIHR fellowship. CC-G was part funded by the NIHR Applied Research Collaboration West Midlands. DA and MP were funded by the NIHR Greater Manchester Patient Safety Translational Research Centre (PSTRC-2016-003).

## Conflict of interest

The authors declare that the research was conducted in the absence of any commercial or financial relationships that could be construed as a potential conflict of interest.

## Publisher's note

All claims expressed in this article are solely those of the authors and do not necessarily represent those of their affiliated organizations, or those of the publisher, the editors and the reviewers. Any product that may be evaluated in this article, or claim that may be made by its manufacturer, is not guaranteed or endorsed by the publisher.

## Author disclaimer

The views expressed are those of the authors and not necessarily those of the NIHR or the Department of Health and Social Care.
