# Supplementary material for: Investigating the links between diagnostic uncertainty, emotional exhaustion, and turnover intention in General Practitioners working in the United Kingdom
Source: Front Psychiatry. 2022 Jul 26;13:936067. doi: 10.3389/fpsyt.2022.936067 (PMC9360551; doi:10.3389/fpsyt.2022.936067)
Supplement: Supplementary file 1 [file Table_1.DOCX]

1. **What is your age?**

|  |
| --- |

1. **What is your full-time (FTE%) equivalent ?**

|  |
| --- |

1. **What is the full-time equivalent (FTE%) of all GPs in your practice?**

|  |
| --- |

1. **How often you feel burned out from your work^1^?**

| Never | A few times a year or less | Once a month or less | A few times a month | Once a week | A few times a week | Every day |
| --- | --- | --- | --- | --- | --- | --- |

1. **How often you feel that you have become callous toward people since you took this job^1^?**

| Never | A few times a year or less | Once a month or less | A few times a month | Once a week | A few times a week | Every day |
| --- | --- | --- | --- | --- | --- | --- |

1. **Have you gone to work with an illness in a situation where you would have recommended a patient to stay home during the last 12 months?^2^**

| None | Once | Two to four  times | More than five times |
| --- | --- | --- | --- |

1. **Your work schedule leaves you enough time for your personal/family life^3^**

| Strongly agree | Agree | Neutral | Disagree | Strongly disagree |
| --- | --- | --- | --- | --- |

1. **In the past year, about what percentage of your patients did you consider difficult to diagnose?^4^**

| 0% | 1% to 5% | 6% to 10% | 11% to15% | >15% |
| --- | --- | --- | --- | --- |

1. **You are satisfied with your career in general practice: ^5^**

| Strongly agree | Agree | Neutral | Disagree | Strongly disagree |
| --- | --- | --- | --- | --- |

1. **What is the likelihood you will leave direct patient care within five years?**

| None | Slight | Moderate | Considerable | High |
| --- | --- | --- | --- | --- |

1. West CP, Dyrbye LN, Sloan JA, Shanafelt TD. Single item measures of emotional exhaustion and depersonalization are useful for assessing burnout in medical professionals. *J Gen Intern Med.* 2009;24(12):1318-1321.

2. Thun S, Fridner A, Minucci D, Løvseth LT. Sickness present with signs of burnout: The relationship between burnout and sickness presenteeism among university hospital physicians in four European countries. *Scandinavian Psychologist.* 2014;1.

3. Shanafelt TD, Boone S, Tan L, et al. Burnout and Satisfaction With Work-Life Balance Among US Physicians Relative to the General US Population. *Arch Intern Med.* 2012;172(18):1377-1385.

4. Sarkar U, Bonacum D, Strull W, et al. Challenges of making a diagnosis in the outpatient setting: a multi-site survey of primary care physicians. *Bmj Qual Saf.* 2012;21(8):641-648.

5. Neumann JL, Mau L-W, Virani S, et al. Burnout, Moral Distress, Work–Life Balance, and Career Satisfaction among Hematopoietic Cell Transplantation Professionals. *Biology of Blood and Marrow Transplantation.* 2018;24(4):849-860.
